# Laboratory Tools to Predict CFTR Modulator Therapy Effectiveness and to Monitor Disease Severity in Cystic Fibrosis

**DOI:** 10.3390/jpm14010093

**Published:** 2024-01-13

**Authors:** Mafalda Bacalhau, Mariana Camargo, Miquéias Lopes-Pacheco

**Affiliations:** 1 Biosystems & Integrative Sciences Institute (BioISI), Faculty of Sciences, University of Lisbon, 1749-016 Lisbon, Portugal; mrbacalhau@fc.ul.pt; 2 Department of Surgery, Division of Urology, Sao Paulo Federal University, Sao Paulo 04039-060, SP, Brazil

**Keywords:** airway cells, bioassay, biomarkers, extracellular vesicles, inflammation, microRNA, precision medicine, organoids, theratyping

## Abstract

The implementation of cystic fibrosis (CF) transmembrane conductance regulator (CFTR) modulator drugs into clinical practice has been attaining remarkable therapeutic outcomes for CF, a life-threatening autosomal recessive genetic disease. However, there is elevated CFTR allelic heterogeneity, and various individuals carrying (ultra)rare CF genotypes remain without any approved modulator therapy. Novel translational model systems based on individuals’ own cells/tissue are now available and can be used to interrogate in vitro CFTR modulator responses and establish correlations of these assessments with clinical features, aiming to provide prediction of therapeutic effectiveness. Furthermore, because CF is a progressive disease, assessment of biomarkers in routine care is fundamental in monitoring treatment effectiveness and disease severity. In the first part of this review, we aimed to focus on the utility of individual-derived in vitro models (such as bronchial/nasal epithelial cells and airway/intestinal organoids) to identify potential responders and expand personalized CF care. Thereafter, we discussed the usage of CF inflammatory biomarkers derived from blood, bronchoalveolar lavage fluid, and sputum to routinely monitor treatment effectiveness and disease progression. Finally, we summarized the progress in investigating extracellular vesicles as a robust and reliable source of biomarkers and the identification of microRNAs related to CFTR regulation and CF inflammation as novel biomarkers, which may provide valuable information for disease prognosis.

## 1. Introduction

Inherited in an autosomal recessive pattern, cystic fibrosis (CF) is a life-threatening progressive disease affecting over 100,000 people worldwide [1,2]. The disease occurs due to mutations in the gene encoding the CF transmembrane conductance (CFTR) protein [3,4,5], a chloride/bicarbonate channel expressed at the apical plasma membrane (PM) that plays a vital role in regulating fluid and ion movements across several epithelial tissues, including lungs, intestine, pancreas, and sweat glands [1,6]. However, despite the multiorgan features of the disease, the elevated morbidity and mortality of people with CF (PwCF) are linked to an accelerated decline of lung function due to repeated cycles of airway mucus accumulation, chronic inflammation, and persistent infection that lead to tissue remodeling and respiratory failure [6,7].

CF diagnosis is based on symptomatology consistent with the disease and laboratory biomarkers that provide evidence of CFTR dysfunction [8,9]. Most new cases of CF are nowadays identified within newborn screening programs by assessing immunoreactive trypsinogen in bloodspots. To confirm the diagnosis, CFTR (dys)function should be demonstrated by: (i) identification of CFTR pathogenic variants in both alleles by genetic analysis, and (ii) elevated sweat chloride concentration (SCC; >60 mmol·L^−1^), altered transepithelial nasal potential difference (NPD) and/or intestinal current measurement (ICM) [8,9]. Although SCC is the standard test for CF diagnosis, the alternative biomarkers are particularly relevant in cases in which clinical signs and symptoms are suggestive of CF, but SCC is intermediate (30–59 mmol·L^−1^) [8,9].

Over 2100 variants have been identified in the CFTR gene [10], of which approximately one-third are now classified as CF-causing [11]. The deletion of a phenylalanine at position 508 (p.Phe508del, legacy: *F508del*) is the most prevalent CF-causing variant, accounting for approximately 70% of all CF alleles [1], while the remaining 30% of CF alleles are represented by an enormous number of CFTR variants and most are (ultra)rare, occurring among few PwCF worldwide [11]. Due to such CFTR allelic heterogeneity, distinct CF phenotypes exist—on average, PwCF with pancreatic insufficient exhibit more severe forms of the disease, while milder phenotypes are usually associated with pancreatic sufficiency [12]. Indeed, these variants cause distinct primary defects, comprising CFTR mRNA and protein biosynthesis, anion transport, and/or PM turnover. Therefore, they have been separated into CFTR variant classes, which are characterized by alterations in (I) expression, (II) folding and trafficking, (III) gating, (IV) conductance, (V) abundance, and (VI) PM stability [13,14]. Overall, CFTR variants in classes I and II are associated with a minimal (or null) function, while a residual (or some) function is usually observed in those variants in classes IV–VI. This grouping offers the advantage that CFTR variants with similar defects might be tackled by similar therapeutic strategies—i.e., theratyping [15].

Over the last two decades, precision (or personalized) medicines targeting the fundamental cause of CF have been developed with tremendous accomplishments attained [14,16]. Indeed, four CFTR modulator drugs are now approved for clinical use: the correctors lumacaftor (LUMA, VX-809), tezacaftor (TEZA, VX-661), and elexacaftor (ELX, VX-445), which retrieve CFTR folding and trafficking to the PM, and the potentiator ivacaftor (IVA, VX-770), which enhances CFTR channel open probability. These drugs—particularly the ‘highly effective’ ones—have provided impressive clinical benefits, representing thus a new dawn for PwCF with eligible genotypes [17,18,19,20,21,22,23]. However, there is a significant number of PwCF carrying (ultra)rare variants for whom no modulator therapy has been approved [11,16].

While traditional clinical trial designs are fundamental in assessing the safety and efficacy of new drugs, and have been feasible for certain subgroups of PwCF—for instance, those homozygous or heterozygous for p.Phe508del [18,19,20,21,22]—these are impractical (and underpowered) for (ultra)rare CF genotypes due to the reduced number of individuals, which are likely to live in different regions worldwide. As a solution to overcome this barrier, translational model systems have been developed based on the own cells/tissue of PwCF and these can recapitulate multiple features of parental organs, thus being used to predict the effectiveness of CFTR modulator drugs at an individual level [24,25,26]. Assessments on these models may provide a feasible starting point for the subsequent clinical testing of the best therapeutic option in alternative clinical studies, such as N-of-1 trials [27,28]. Moreover, because CF causes chronic airway inflammation, related biomarkers should be integrated into routine care to continuously monitor disease status and progression as well as CFTR modulator effectiveness.

In this review, we have focused on the utility of novel in vitro tools to assess CFTR modulator responses and their ability to provide prediction of in vivo therapeutic effects. Aiming to monitor CF severity and progression, studies investigating the impact of CFTR modulator therapies on inflammatory biomarkers have also been discussed. Finally, advances in the development of novel laboratory biomarkers (namely, extracellular vesicles and microRNAs) have been summarized.

## 2. Laboratory Tools to Predict CFTR Modulator Effectiveness In Vivo

**Figure 1:**
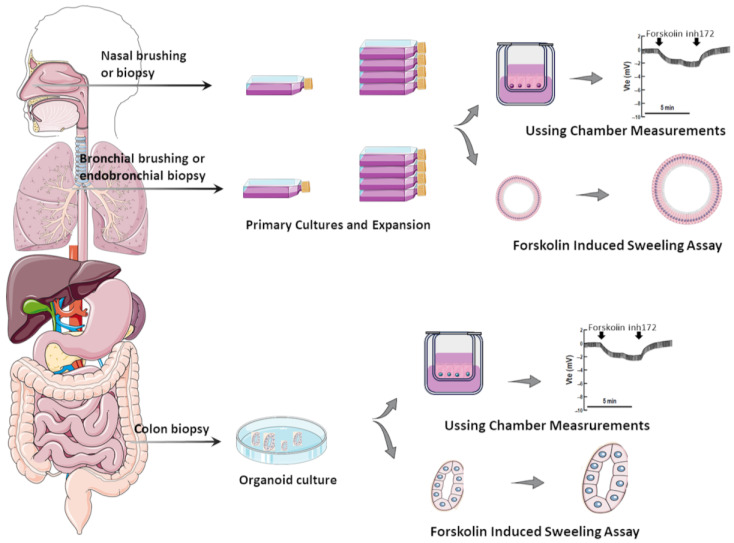
**Translational human CF model systems for personalized/precision medicine.** Airway samples can be collected from the nose or bronchi by brushing or biopsies and gastrointestinal samples can be collected from the colon or rectum by biopsies. These samples are cultured in specific in vitro conditions, expanded, and then seeded on porous membranes to grow in polarized monolayers or in matrigel to form organoids/spheroids. Cell monolayers and organoids can be used to assess CFTR function/rescue through Ussing chamber measurements and through forskolin-induced swelling assays, respectively.

Several in vitro assays have been developed using various model systems to comparatively assess the efficacy of CFTR modulators (individually or combinations thereof) [29]. Although heterologous cell lines have been fundamental to enhance the understanding of CFTR biology at genetic, biochemical, and physiological levels, in the context of precision medicine, they can only be used for variant theratyping (i.e., matching single variants to modulators), being unable to predict responses of a determined individual to a specific therapy. Accordingly, alternative translational models have been established by using primary cells from PwCF to inquire about responses at an individual level (Figure 1).

While cells heterologously expressing CF-causing variants remain very useful for CFTR studies, primary cell models can provide a more sensitive and reliable prediction of therapeutic responses for several reasons: (i) The last express CFTR in the native genomic context, while cell lines frequently use CFTR cDNA (a copy of the mature mRNA, which lacks the introns); therefore, cells lines may not recapitulate certain cellular mechanisms, including nonsense-mediated decay or splicing effects. For instance, p.Gly970Arg (legacy *G970R*) was thought to be a CFTR gating variant based on cDNA expression findings [30,31]; however, analysis of cells from PwCF carrying this variant revealed that it actually causes a splicing defect [32,33]. (ii) The cellular background has a marked influence on CFTR processing and function as well as its pharmacological sensitivity. As exemplified by the cases of p.Phe508del [34,35] and p.Gly1244Glu [36], the complexity of cellular processes related to protein biogenesis and folding, as well as its PM trafficking, may not be completely recapitulated in cell lines—particularly those from non-human and/or non-respiratory epithelium origin [29]. Likewise, CFTR gene regulation can be impacted by epigenetic factors and these are only taken into account by the assessment in primary cells from each individual [37]. (iii) Characterization of single variants can be efficiently accomplished in cell lines; however, two variants indicating to be low responsive (below therapeutic relevant threshold) in separated cell lines can compose a genotype with a good prediction for clinical benefits (if evidenced by assessing responses in this individual’s cells). This is particularly relevant for (ultra)rare CFTR variants that are frequently identified in racial and ethnic minority populations, which are usually excluded from traditional clinical trial designs [38]. 

Moreover, numerous reports have established correlations of the data on primary cell models with clinical features of PwCF (before and after initiating modulator therapy) to provide a translational perspective of therapeutic effects (Table 1). Accordingly, these can serve as potential biomarkers to identify which drug(s) could be the best therapeutics for every individual with CF—i.e., “the right therapy for the right person”.

### 2.1. Primary Airway Cells Grown in Monolayers

Since the development of the first CFTR modulators, primary human bronchial epithelial (HBE) cells have been considered the gold standard to confirm the efficacy of these drugs in vitro for the subsequent clinical assessment [60,61,62]. These cells can be obtained either from the lungs of individuals undergoing transplant or by bronchial brushing. However, despite the development of well-established protocols to expand and maintain HBE cells to high passage numbers [63], bronchoscopy is a considerably invasive procedure that requires sedation and anesthesia. Likewise, the need for explanted lungs limits the availability of these cells, particularly of (ultra)rare CF genotypes.

Such limitations were overcome by the adoption of a method of conditional reprogramming of cells [64,65,66] and the usage of cells from the nasal epithelium [39,67], which have become routinely used by several CF research groups. Nasal epithelial cells can be obtained through minimally invasive procedures, such as nasal brushing or scraping of the lower turbinates [65,67], which is well tolerated by children and adults with CF and does not require sedation or anesthesia. When cultured under conditional reprogramming conditions, human nasal epithelial (HNE) cells acquire progenitor stem-cell-like features, enabling their expansion with prolonged lifespan and differentiation into various cell types of the respiratory tract [40,65,66,68,69]. Although epithelial cell populations can be distinct in the upper and lower airways [70,71,72], studies comparing HNE and HBE cells differentiated at the air–liquid interface (ALI) demonstrated that they exhibit similar morpho-functional properties and response to inflammatory cytokines [39,48,73]. HBE and HNE cells from the same individual also demonstrated equivalent CFTR-mediated anion transport in electrophysiological measurements [25,39]. The analysis of CFTR function in these cells relies primarily on the bioelectric movement of ion transport assessed in micro-Ussing chambers or patch clamps [74,75]. It is notable that both HBE and HNE cells are highly sensitive to culture conditions [63]; therefore, it is imperative to standardize protocols and reference cells to ensure reproducibility among different operators and laboratories. 

Several reports have indicated that HNE cells can be successfully used as a surrogate for HBE cells in CFTR studies and theratyping [25,39,40]. It is notable that strong correlations were described in the in vitro rescue of CFTR function by modulator drugs in both cell types and in vivo alterations in SCC [39]. Data from IVA-promoted CFTR-mediated chloride transport in HNE cells also correlated well with alterations in SCC and ppFEV_1_ of PwCF carrying either p.Arg117His (legacy: *R117H*) or p.Gly551Asp (legacy: *G551D*) [41]. Responses of CFTR function to modulator drugs in HNE cultures also demonstrated a good correlation with alterations in SCC, ICM, and lung function (measured as percent predicted forced expiratory volume in one second [ppFEV_1_]) of PwCF carrying rare genotypes [40,43,44,45,76] or homozygous for p.Phe508del [25,77]. Furthermore, modulator-promoted responses in HNE cultures have been assessed to identify non-eligible responders for compassionate use [45]. Altogether, these studies indicate that measuring CFTR function in HNE cultures serves as a good predictor of clinical benefits that can be subsequently verified in vivo to enhance the access of CFTR modulator drugs for PwCF carrying common and rare variants.

### 2.2. Airway Organoids/Nasospheroids

Because the primary assessment of CFTR function in HBE and HNE cultures is based on micro-Ussing chamber measurements, which is a low-throughput technique, protocols have been optimized to culture these cells into 3D models [26,47,49,78], allowing thus for the assessment of CFTR function in high throughput. Initial studies found that these 3D models can recapitulate various features of the in vivo airway epithelia, including expression of tight junctions, cilia, and mucins [79], and assessment of CFTR-mediated fluid secretion on airway organoids enables to discriminate CF and non-CF cultures [26,47]. Furthermore, CFTR-mediated chloride transport in micro-Ussing chamber measurements of HNE cultures were found to closely correlate with forskolin-induced swelling (FIS) assay of airway organoids. The latter is a microscopy-based functional assay in which CFTR function can be indirectly measured based on fluid movement upon CFTR stimulation by forskolin. When CFTR is activated/rescued, an increase in organoid size/swelling occurs [29,80].

Airway organoids can be generated in two configurations [26,49]: (i) With the apical membrane located at the inside due to the presence of a physical matrix (e.g., matrigel) in the culture. In this case, CFTR activation leads to organoid swelling, since fluid secretion occurs from the basal to the apical side. (ii) On the other hand, the omission of a physical matrix in the culture enables the formation of organoids with the apical membrane located outside, and CFTR activation leads thus to organoid shrinking [26,49]. For the in vitro assessment of CFTR function/rescue, the first configuration and the FIS assay have been the most broadly employed recently [29,49,80].

Upon rescue of CFTR using modulator drugs, responses were demonstrated in organoids from PwCF carrying p.Phe508del in both alleles [26,47] or a range of rare CFTR variants [48,49]. Furthermore, CFTR baseline and modulator-rescued responses in airway organoids demonstrated a significant correlation with alterations in SCC and ppFEV_1_ [48]. Despite such progress, further development and refining of airway organoid technology is needed, since greater variability in results was reported as compared to those of HBE and HNE cells in ALI cultures [26,47]. Airway organoids also exhibited CFTR-independent swelling that was promoted by the stimulation of alternative ion channels [49].

### 2.3. Intestinal Organoids

Among PwCF, intestinal organoids have been frequently obtained from rectal biopsies, which is a relatively invasive procedure but one which is well tolerated by individuals [80]. From these samples, LGR5^+^ adult stem cells from intestinal crypts are isolated and cultured in a physical matrix—the most broadly used is matrigel—with a specific medium containing appropriate growth factors that enable their stemness maintenance for the expansion and self-organization into 3D structures termed organoids [81,82]. These cells can thus be cultured and expanded for long periods without losing their ability for self-renewal and growth. They can thus be used for the assessment of currently available modulators or be biobanked for future studies. Intestinal cells also have higher CFTR expression levels compared with airway cells [83], such as HBE and HNE cells, which offers an advantage for CFTR studies.

Similar to airway organoids, the FIS is the most used assay for the assessment of CFTR modulators in intestinal organoids [80]. Since CFTR is active in healthy individuals, their organoids have a rounder shape, with a fluid-filled, steady-state lumen, under basal culture conditions. On the other hand, organoids from PwCF have a more irregular aspect with less visible lumen. Such differences led to the development of two scoring criteria for evaluating differences in organoid morphology: (i) the steady-state lumen area (SLA) [24,80] and (ii) the rectal organoid morphology analysis (ROMA) [84]. The SLA measures and compares the lumen area with the total organoid area, and is expressed as the percentage of the total organoid area [24,80]. The ROMA assesses the circularity index, which measures the roundness of the organoids, and the intensity ratio, which measures the presence/absence of a central lumen [84]. By using these parameters, both SLA and ROMA were able to discriminate between organoids of healthy individuals and PwCF, as well as CFTR rescue by modulator drugs [24,80,84].

Results from FIS of intestinal organoids demonstrated good correlations with responses in other samples from the same individual, namely current measurements in rectal biopsies and HNE cells [48,85]. Other studies have also demonstrated a strong correlation between FIS of intestinal organoids with SCC [24,50], which enabled the stratification of children with CF based on the disease severity [51]. Furthermore, ppFEV_1_ and body mass index presented consistent correlations with FIS of intestinal organoids [52,56]. Regarding CFTR modulators in PwCF, various studies demonstrated that the FIS assay of intestinal organoids can be a feasible biomarker for predicting clinical benefits [24,53,57]. Indeed, a high correlation between modulator-promoted responses in intestinal organoids and alterations in SCC and ppFEV1 in PwCF carrying common and rare variants has been reported in several studies [24,50,53]. Moreover, modulator-promoted responses in intestinal organoids have served as a basis for guiding eligibility for compassionate use and to obtain health insurance coverage for individuals carrying non-eligible responsive CFTR variants [54,58].

Altogether, these reports confirm the high throughput of organoids and support their use as a valuable tool for precision medicine approaches. However, limitations should be considered as these organoids are derived from intestinal cells and thus may not completely recapitulate airway/lung biology. Furthermore, although FIS of intestinal organoids appears to be very sensitive to CFTR function (even in a low functional range), assessments are limited to the structure stretching, which might underestimate high responses due to assay ceiling effects. Finally, organoids from healthy individuals are already pre-swollen in baseline conditions, indicating that FIS might also be underestimated in organoids of PwCF-carrying variants with high residual function.

## 3. Laboratory Biomarkers to Monitor Lung Disease Progression

In vivo CFTR biomarkers, such as SCC and NPD, have been routinely used in CF diagnoses [8] with studies also demonstrating the potential correlation of these measurements with clinical features [86,87,88], including lung function tests (FEV_1_ or lung clearance index) and body mass index. Furthermore, some studies have used SCC and NPD as parameters to assess the therapeutic effects of CFTR modulators [17,20,21,22]. However, inherent limitations to SCC and NPD exist, including responses influenced by secondary effects [89], considerable inter- and intra-individual variability [90,91], and the inability to accurately distinguish disease severity [87,88].

**Figure 2:**
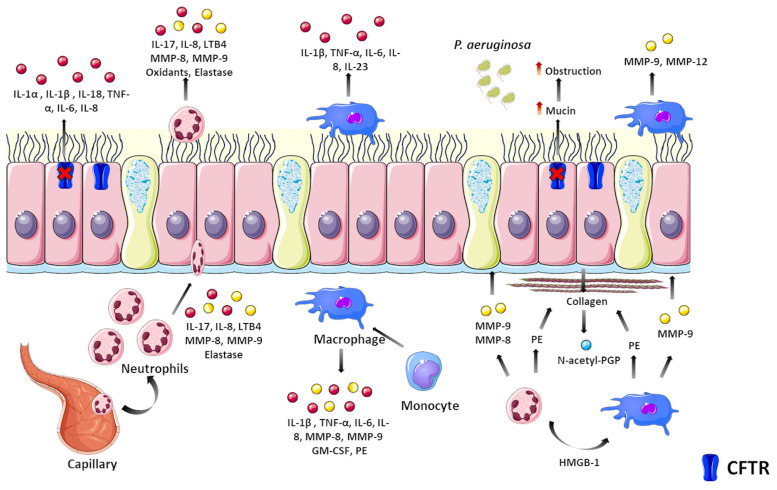
**Pathological events in the CF airway inflammation.** Loss of CFTR activity in airway cells induces abnormal cytokine signaling, including upregulation of nuclear factor (NF)-κB with subsequent increased production and secretion of pro-inflammatory mediators. These cytokines/chemokines induce neutrophil activation and transmigration into the airways to secrete oxidants, proteases, and other inflammatory mediators that damage airway cells. Such a process can be further intensified by the presence of pathogens, such as *Pseudomonas aeruginosa*, due to lipopolysaccharide (LPS) that acts as a pathogen-associated molecular pattern. Monocytes and macrophages also contribute to the hyperinflammatory state by secreting cytokines/chemokines that lead to further recruitment of inflammatory cells. Damaged cells are replaced by the deposition of extracellular matrix components, such as collagen, which elicits neutrophil chemotaxis and tissue remodeling with loss of function. Abbreviations: GM-CSF—granulocyte–macrophage colony-stimulation factor; HMGB—high-motility group box; IL—interleukin; LTB—leukotriene; MMP—metalloprotease; PE—prolylendopeptidase; TNF—tumor necrosis factor.

Because PwCF present a chronic inflammatory process in their lungs, cytokine/chemokine production remains continuously active, leading to overexpression of pro-inflammatory mediators and potential downregulation of several anti-inflammatory/resolving factors [92,93]. Indeed, various pro-inflammatory cytokines/chemokines have been found in elevated levels in PwCF, but few of these biomarkers have been routinely used in the clinic. A brief overview of CF airway inflammation is depicted in Figure 2.

Biomarkers of airway inflammation have been mainly detected through assessing serum blood, bronchoalveolar lavage fluid (BALF), and sputum (induced or spontaneously expectorated). Detection of single and multiple biomarkers has been investigated to potentially enhance current models of disease progression and to monitor the clinical effects of CFTR modulator therapies. Below, we have included studies investigating the impact of clinically approved modulators on pro-inflammatory biomarkers (Table 2).

### 3.1. Monitoring Ivacaftor Effects on CF Inflammation

In a study assessing inflammatory mediators in the sputum of PwCF, a significant reduction in levels of interleukin (IL)-8, IL-1β, and neutrophil elastase (NE) was observed since the first week of IVA treatment and sustained for the 2-year follow-up [97]. Such an effect was also accompanied by a decrease in the relative abundance of *Pseudomonas aeruginosa*, *Burkholderia* sp., and *Staphylococcus aureus* in the sputum of these participants. In addition, mass spectrometry analysis revealed a decrease in the levels of other relevant mediators, namely arginase-1, myeloperoxidase, calprotectin, and S100A9 [97]. Nevertheless, results from other reports demonstrated a decrease in the relative abundance of *P. aeruginosa* after IVA initiation without altering levels of inflammatory mediators [94,98,113], while two additional studies did not observe differences in levels of IL-8, IL-1β, NE, or alpha 1-antitrypsin nor bacterial load in sputum of PwCF carrying p.Gly551Asp taking IVA [95,99]. Likewise, no differences in the levels of IL-8 and free NE and neutrophil count were found in BALF of preschool children with CF before and after IVA initiation [103]. 

The impact of IVA initiation was assessed in blood samples from PwCF by analyzing whole proteome and specific mediators [114]. A reduction in the levels of calprotectin, a neutrophilic marker, and high-mobility group box 1 (HMGB-1), a pro-inflammatory mediator, was observed after one month of IVA and sustained for the 6-month follow-up. However, no changes were found in levels of C-reactive protein (CRP), IL-13, or IgG before and after IVA initiation. Reactome pathway analysis revealed changes in protein amounts related to plasma lipoprotein assembly, remodeling, and clearance, and extracellular matrix organization after IVA treatment [114]. In other reports, IVA initiation was able to decrease levels of IL-6, IL-8, IL-10, CRP, and white blood cell count [98,101], although tumor necrosis factor (TNF)-α levels and mononuclear cell count remained unaltered [96,98]. The effects of IVA on CF inflammation were also investigated in epithelial lining fluid obtained from the nasal lavage of participants before and after treatment initiation [100]. Although only 10 PwCF were enrolled in this study, a reduction in levels of IL-1β, IL-6, and IL-8, but not NE, was observed after 8-12 weeks of IVA treatment [100].

### 3.2. Monitoring Lumacaftor/Ivacaftor and Tezacaftor/Ivacaftor Effects on CF Inflammation

While IVA treatment demonstrated significant clinical benefits for PwCF carrying at least one gating/conductance variant in clinical trials [17,115,116,117], the therapeutic effects of LUMA/IVA were rather very modest on p.Phe508del-homozygous individuals [18]. Only a few studies have investigated the impact of LUMA/IVA on the CF inflammatory process and the findings are controversial [104,105]. In 14 PwCF taking LUMA/IVA, a decrease in SCC was observed, but no differences in white blood cell count and CRP values in the 12-month follow-up [104]. Nevertheless, blood-derived mononuclear cells from these participants were isolated and demonstrated an increase in chloride efflux after LUMA/IVA treatment, suggesting a restoration of CFTR protein function [104]. In another study, 75 PwCF were enrolled at the initiation of LUMA/IVA and 41 were able to spontaneously produce sputa before and 6 months after treatment [107]. Although a significant improvement in body mass index and the need for intravenous antibiotics were observed in the 6-month follow-up, no changes in *P. aeruginosa* relative abundance and calprotectin values occurred [107]. On the other hand, another study evidenced a significant decrease in inflammatory biomarkers in the blood (IL-1β, IL8, and TNF-α) and sputum (IL-1β, IL-6, IL-8, and TNF-α) for PwCF taking LUMA/IVA for 12 months [105]. These findings are in line with the improvement in lung function tests (ppFEV_1_ and lung clearance index), reduction in bacterial load and IL-1β in sputum, and increase in Shannon diversity of the airway microbiome of 30 PwCF taking LUMA/IVA for 8-16 weeks [106].

Similar to LUMA/IVA, the therapeutic effects of TEZA/IVA on p.Phe508del-homozygous individuals were modest, but the last was also associated with fewer adverse effects [20]. In CF monocytes in vitro, both LUMA/IVA and TEZA/IVA were able to reduce IL-18 values, but IL-1β levels only reduced with TEZA/IVA treatment [108]. Thirteen adults with CF taking LUMA/IVA and eight taking TEZA//IVA were followed for 3 months and the effects of treatment on inflammatory responses were assessed. A significant decrease in serum IL-18 and TNF values was observed by both treatments; however, only TEZA/IVA treatment resulted in the reduction in IL-1β levels [108]. 

### 3.3. Monitoring Elexacaftor/Tezacaftor/Ivacaftor Effects on CF Inflammation

In comparison to LUMA/IVA and TEZA/IVA [18,20], the triple combination ELX/TEZA/IVA attained greater clinical benefits for PwCF carrying at least one p.Phe508del allele in clinical trials [21,22], with its safety and efficacy also being assessed in several case reports [23]. Such remarkable restoration of CFTR function led to the US FDA approval of this combinatorial therapy to many (ultra)rare variants based on findings on heterologous cell lines [16,118]. Furthermore, additional clinical investigations have been pursued to assess the effects of ELX/TEZA/IVA treatment in other aspects of the disease [23], including the CF inflammatory process.

In 48 PwCF taking ELX/TEZA/IVA for 6 months, there was a significant reduction in the presence of *P. aeruginosa* and *S. aureus* with 30% of negative cultures associated with improved ppFEV_1_ [109]. Furthermore, ELX/TEZA/IVA treatment decreased serum levels of IL-6, IL-8, and IL-17A and normalized white blood cell count and composition [109]. Likewise, lung and systemic inflammation were assessed by analyzing blood and spontaneously expectorated sputum of 30 PwCF before and after 3 and 12 months of ELX/TEZA/IVA treatment [112]. Significant reductions in the activity of NE, proteinase 3, and cathepsin G were observed in the 3-month follow-up. In line with other reports [110,111], *P. aeruginosa* relative abundance was accompanied by reduced levels of IL-1β and IL-8 in sputum [112]. ELX/TEZA/IVA treatment in PwCF with advanced disease also led to a reduction in plasma levels of IL-6, CRP, and soluble TNF receptors [112]. Although ELX/TEZA/IVA treatment significantly improved mucus viscoelastic properties and reduced levels of inflammatory mediators, the values did not reach those of the healthy controls in a 12-month follow-up [109]. Altogether, in addition to the clinical benefits promoted by the triple combinatorial therapy, it demonstrated anti-inflammatory properties, which may contribute to improved clinical outcomes from short- and long-term perspectives.

## 4. Novel Laboratory Tools to Assess CF Status and Progression

Despite the fact that several biomarkers exist to assess CF severity and progression, some are associated with invasive procedures (e.g., bronchoscopy to obtain BALF), which limit their utility in routine care [119,120]. As an alternative, sputum biomarkers have been used and represent a minimally invasive way to assess airway inflammation and infection [119,121,122]. However, because ‘highly effective’ modulator therapies may significantly decrease sputum production, obtaining such samples may become more challenging. Moreover, disease signs and symptoms may become less evident early in life with the earlier initiation of modulator therapies. There is thus a need to identify novel, robust, and sensitive biomarkers to monitor disease progression, even before symptoms onset. Among the various biomarkers in development to predict clinical changes in CF, we have discussed herein the assessments of extracellular vesicles (EVs) as a robust source of biomarkers and the identification of microRNAs related to CFTR regulation and CF inflammation. 

### 4.1. Extracellular Vesicles

In recent years, EVs have been intensively investigated not only as potential treatments [123,124] but also as suitable sources of biomarkers for assessing disease states [125]. These consist of lipid-membrane-bound vesicles that range between 0.03 and 5.0 µm in diameter and contain bioactive cargoes, including proteins, lipids, DNA, mRNAs, and microRNAs, among others; these reflect the physiologic or pathologic state of parental cells [125]. Because EVs are a heterogeneous population of particles, they have been grouped into three main types based on their size and subcellular origin: exosomes, microvesicles (or microparticles), and apoptotic bodies. Exosomes are the smallest EVs (0.03–0.15 µm) and are limited by a phospholipid layer formed by the fusion of late endosomes and the PM, being thus released into the extracellular space via exocytosis. Microvesicles range between 0.15 and 1.0 µm in diameter and are derived from outward budding and scission of the PM. Finally, apoptotic bodies are the largest EVs (1.0–5.0 µm) and originated from the apoptotic process of cells. They can contain cellular organelles, DNA and RNA fragments, and other cytosolic components and are characterized by the exposure of phosphatidylserine to the outer side [125]. Although the biogenesis of EVs is different, their size and density can overlap, which makes it difficult to isolate a pure EV population with the current technique available. Nevertheless, it is recognized that EVs play a critical role in inter- and intracellular communications, acting as both paracrine and endocrine signals [126]. 

EVs can be secreted by virtually all cell types and are present in various biological fluids, such as blood, sputum, BALF, and urine. Although EV-transported cargoes are cell-specific, they are also influenced by the source from which EVs originated and environmental stimuli that can alter intracellular signaling [127]. Once these particles are released into the extracellular space, they can also affect the phenotype of recipient cells, which makes EV cargoes relevant biomarkers to be used for disease diagnosis and prognosis.

Recent evidence has indicated that EVs participate in the homeostatic regulation of cells throughout the respiratory tract [123,128,129,130,131] and can be involved in the pathological mechanisms of chronic inflammation in asthma, chronic obstructive pulmonary disease, and CF [127,132,133,134]. Indeed, a growing number of studies have demonstrated that EVs derived from CFTR-mutated cell lines or samples from PwCF can induce neutrophil chemotaxis and, in turn, neutrophil-derived EVs can stimulate the activation of inflammasome in airway epithelial cells [132,135,136,137,138]. In spontaneously expectorated sputum from 21 PwCF, a greater number of EVs—mainly microvesicles—were observed in comparison with sputum collected from 7 patients with primary ciliary dyskinesia [132]. Sputum-derived EVs from PwCF were predominantly of granulocyte origin and when these were intratracheally administered in mice, they promoted pro-inflammatory effects. Interestingly, the administration of EVs from PwCF caused a granulocyte-dominated peribronchial and perivascular inflammation, while a macrophage-predominant inflammation was observed by the administration of EVs from patients with primary ciliary dyskinesia [135]. Proteomics was also performed to assess the content of BALF-derived EVs from PwCF, patients with primary ciliary dyskinesia and asthma [136]. In this analysis, a higher amount of proteins related to antioxidant activity and leucocyte chemotaxis was found in EVs from PwCF and patients with primary ciliary dyskinesia compared to those from patients with asthma [136].

In an in vitro study, EVs from non-CF (16HBE14o^−^, NuLi-1) and CF cell lines (CFBE41o^−^, CuFi-5) were isolated and analyzed. A significantly higher number of EVs was released from CF cell lines compared to non-CF ones [137]. CFBE41o^−^ cell-derived EVs also exhibited increased content of proteins related to neutrophil recruitment and degranulation. When these cells were treated with LUMA/IVA or TEZA/IVA, there was a reduction in the number of EVs released. Furthermore, differences in protein expression content were observed in BALF-derived EVs from PwCF of different age groups and between PwCF and healthy controls [137]. More recently, EVs were isolated from CF airway fluid and demonstrated to be enriched with IL-1α, IL-1β, IL-18, and active caspase-1 [138]. These are mainly neutrophil-derived EVs and are able to induce primary granule exocytosis and activate caspase-1 and IL-1β production, stimulating thus a hyperinflammatory state. In this context, activated neutrophil-derived EVs induced the upregulation of active caspase-1 in primary tracheal cells, promoting inflammasome activation in neighboring cells [138].

It is expected that sputum production from PwCF will significantly reduce with the increasing number of individuals taking ‘highly effective’ modulator therapies. Aiming to assess alternative options, a recent study investigated the potential utility of serum-derived EVs for further diagnosis investigations [139], since EVs can be transported by the bloodstream upon release from parental cells. Although no differences were observed in EV numbers between children with CF and controls, a significantly greater number of serum-derived EVs was found in adults with CF. Nevertheless, 12 months of ELX/TEZA/IVA treatment significantly altered EV-carried proteins in PwCF from the different age groups [139]. Altogether, these findings indicate the feasibility of using EVs derived from blood or samples from the airways to monitor CF disease severity and the therapeutic effects of modulators. Nevertheless, further standardization of EV isolation and characterization methods are vital to provide robust data regarding their relevance as biomarkers and for use in the clinic.

### 4.2. MicroRNAs

MicroRNAs (or miRs) are small RNA molecules that function as negative regulators of RNA transcriptions in various biological processes [140]. They have been reported to promote the degradation of targeted mRNA, thus decreasing or even abrogating biosynthesis and function of specific proteins [141]. Indeed, several miRs were demonstrated to be overexpressed in disease states [142] and an increasing number of studies has investigated the participation of miRs in CFTR regulation and CF inflammatory processes (Table 3).

In a study aiming to assess the relationship between CFTR allelic heterogeneity (p.Phe508del-homozygous and -heterozygous) and infection by *P. aeruginosa*, *S. aureus*, and *Aspergillus* sp., three miRs (miR-145, miR-223, and miR-494) were found to be upregulated in cell lines and primary bronchial cells from PwCF [148]. When samples from PwCF were separated based on the bacterial infection, the subgroup that was infected by *P. aeruginosa* exhibited the most significant increase in the expression of these three miRs and a decrease in CFTR activity [148]. Similar findings were observed in other studies, demonstrating the importance of miR-145, miR-223, and miR-494 in the regulation of CFTR function and how they are influenced by bacteria infection [147,149,153]. MiR-145 and miR-494 were also found to regulate SMAD3 expression, which is involved in TGF-β-mediated responses [147]. Indeed, TGF-β has been described as a CF gene modifier and directly affects CFTR function [154] by stimulating miR-145 expression and, consequently, inhibiting CFTR biosynthesis in airway epithelia [150]. MiR-145 and TGF-β mRNA levels were 5- and 10-fold higher, respectively, in BALF-derived EVs from PwCF compared to controls [150].

Because IL-8 is a relevant cytokine/chemokine involved in CF airway pathogenesis, miRs able to modulate this factor have been investigated [143,145,151]. In silico data indicated that miR-17 and let-7b have a role in IL-8 regulation and BALF from PwCF was assessed to validate these findings. Both mRNA and protein levels of IL-8 were significantly higher in CF samples compared to non-CF ones. MiR-17 was demonstrated to post-transcriptionally regulate IL-8 production, although let-7b was not as effective but still played a role at the transcript level [143]. Nevertheless, another study found that let-7b expression was 5-fold higher and played a role in the PI3K/Akt pathway in the IB-3 CF cell lines [151]. Likewise, the PI3K/Akt pathway was modulated by miR-155 [151]. Another possible target for IL-8 is miR-93. In IB3-1, CuFi-1, and NuLi-1 cells infected by *P. aeruginosa*, a decrease in miR-93 expression was associated with the increase in IL-8 production [145]. This study also pointed to miR-494 as a potential target for IL-8 modulation during *P. aeruginosa* infection [145].

Other miRs were also suggested to participate in CFTR regulation and CF inflammatory processes. For instance, miR-31 regulates cathepsin-S, a protease that is involved in lung inflammation by degrading antimicrobial proteins. In CF airway epithelial cells, a significant decrease in miR-31 expression was observed, leading to increased levels of cathepsin-S, which also correlated to changes in expression of the transcription factor IRF-1 [144]. Treatment of cells with miR-31 mimics reduced IRF-1 protein levels and, consequently, reduced cathepsin-S production [144]. MiR-199a-5p, expressed in both human and murine macrophages, was found to participate in CF immune responses by regulating the hyperinflammatory state via the PI3K/Akt pathway [152]. Compared to the control, CF cells exhibit downregulation of miR-126 and upregulation of TOM1, a protein able to bind to a Toll-like protein and thus negatively regulates TLR2, TLR4, IL-1β, and TNF-α [146]. When cells were transfected with a synthetic pre-miR-126, a decrease in TOM1 protein levels was evidenced. On the other hand, TOM1 knockdown led to increased secretion of IL-8 via NF-κB signaling, demonstrating the role of miR-126 in regulating this immune response [146]. 

The Mirc1/Mir-17-92 cluster, which is composed of six miRs involved in autophagy, was found to be highly expressed in a CF mouse model. This cluster inhibits autophagy and improves CFTR function. In samples from PwCF, Mirc1/Mir-17-92 cluster expression was significantly higher in sputum but not in plasma or CF neutrophils [141]. The elevated expression of this cluster in sputum positively correlated with lung function; however, no differences were observed in Mirc1/Mir-17-92 cluster expression in PwCF receiving LUMA/IVA treatment in the 6-month follow-up [141]. On the other hand, nesolicaftor (PTI-428), a CFTR amplifier, was demonstrated to reverse the CFTR inhibition caused by miR-145 mediated by TGF-β signaling in CF cells [155]. MiR-145 antagonism was also able to restore LUMA-rescued p.Phe508del-CFTR processing by blocking miR-145 action and the TGF-β pathway [150]. Altogether, these studies have demonstrated the relevance of miRs in CFTR regulation and CF inflammation, highlighting the feasibility of assessing them to monitor disease progression and modulator therapy effectiveness.

## 5. Concluding Remarks

We are in a new era of CF therapeutics in which ‘highly effective’ modulator drugs are providing life-transforming perspectives for many PwCF worldwide. Several human CF model systems (primary HBE/HNE cells and airway/intestinal organoids, among others) have emerged to assist in the validation of modulator efficacy in vitro. Correlations of clinical data with responses in these translational laboratory tools have also been established to provide a sensitive and reliable prediction of therapeutic effectiveness, aiming to expand the availability of currently approved modulator therapies for non-eligible rare CF genotypes who are responsive. Furthermore, these tools continue to be enhanced and may enable new ways to deliver personalized care in a timely manner.

Despite such accomplishments, novel biomarkers are needed to follow disease status and progression. Several research efforts have been performed to assess the impact of modulator therapies on well-known CF inflammatory mediators. MicroRNAs and EVs have also been investigated as alternative biomarkers and minimally invasive biomarker sources, respectively, for disease prognosis. Detection of a highly sensitive biomarker or a panel thereof will be essential in routine care to enable personalized recommendations in a growing CF population with a more manageable chronic disease—instead of a life-threatening one.

## Figures and Tables

**Table 1:** Studies reporting correlations of CFTR modulator-promoted responses in cell models and clinical effects in PwCF.

Model System	CF Population/Genotypes	CFTR Modulator(s)	Key Findings/Correlations	Ref.
HNE cells	PwCF homozygous for p.Phe508del or carrying genotypes leading to minimal or residual CFTR function	LUMA TEZA IVA	The level of CFTR rescue significantly correlated with the ppFEV_1_ change at 6 months in 8 PwCF treated with CFTR modulators.	[25]
	Ten PwCF (<19 years) with a wide range of CFTR variants	LUMA IVA	CFTR modulation produced changes in CFTR function in nasal and bronchial cell cultures. There was a correlation between the residual CFTR function in both cell types from PwCF with SCC between 60 and 90 mmol·L^−1^.	[39]
	Five healthy subjects (they were not genetically tested) and eight PwCF with variants associated with minimal or residual CFTR function	–	CFTR-mediated transepithelial chloride currents measured in vitro through Ussing chamber assay correlated with the SCC of subjects.	[40]
	Twelve adults with CF with either the p.Gly551Asp or p.ArgR117His	IVA	A strong correlation between changes in SCC and in vitro CFTR activation was observed. Furthermore, a moderate correlation between in vitro CFTR activation and changes in ppFEV_1_ was reported.	[41]
	Seven PwCF (≤16 years, clinically stable) with residual CFTR function variants	IVA	All subjects with decreased SCC in response to IVA treatment also had significant increases in chloride current in HNE cultures with IVA exposure.	[42]
	Healthy volunteers, PwCF with p.PheF508del/p.Arg117His-7T, p.PheF508del/p.PheF508del, p.PheF508del/p.Met284fs, p.Arg334Trp/c.406-1G>A, and p.Ser18ArgfsX16/p.Ser18ArgfsX16	LUMA TEZA IVA	In vitro CFTR chloride measurements correlated to changes in SCC.	[43]
	Five PwCF: one homozygous for p.Ser737Phe and four compound heterozygous for p.Ser737Phe	TEZA ELX IVA	In vitro analysis demonstrated different levels of CFTR activity. Some degree of CFTR dysfunction was detected by evaluating chloride secretion in HNE cells derived from compound heterozygous subjects; CFTR activity was improved by IVA alone and even more by treatment with correctors.	[44]
	Eleven PwCF carrying FDA-eligible variants for ELX/TEZA/IVA and twenty-eight PwCF carrying non-eligible variants	TEZA ELX IVA	There was a significant relationship between CFTR activity correction and changes in ppFEV_1_ or SCC.	[45]
	A 56-year-old male with CF and the p.Phe508del/p.Gln1291His genotype	TEZA ELX IVA	In HNE cells, p.Phe508del/p.Gln1291His resulted in reduced baseline CFTR activity, and showed minimal response to ELX/TEZA/IVA (individually or in combination), aligning with the individual’s clinical evaluation as a non-responder to these drugs.	[46]
Airway organoids	Nine healthy volunteers and three PwCF	LUMA IVA	Organoids treated with modulators displayed similar effects to clinical response; LUMA/IVA treatment promoted greater responses in organoids from p.PheF508del-homozygous subjects.	[26]
	Six healthy volunteers, nine PwCF homozygous for p.Phe508del, and ten with at least one non-p.Phe508del variant	LUMA IVA	p.Phe508del/p.Phe508del organoids treated with LUMA/IVA exhibited a positive change in swelling responses; a relationship between organoid response to drug and in vivo clinical response was observed for three subjects treated.	[47]
	Eighteen PwCF and five healthy volunteers	TEZA ELX IVA	In vitro responses to CFTR modulators correlated well with clinical measurements.	[48]
	Five PwCF: p.Phe508del/p.Phe508del, p.Phe508del/p.Gly542X, and F508del/G542X and p.Arg334Trp/p.Arg334Trp	LUMA IVA	Responses of organoids correlated well with clinical findings.	[49]
Intestinal organoids	Twelve healthy volunteers, four CF carriers (WT/p.Phe508del), thirty-five PwCF carrying class II and III variants, and eighteen PwCF carrying class IV and V variants	LUMA IVA	Responses of organoids to CFTR modulators correlated with outcome data from clinical trials.	[24]
	Twenty-four PwCF: fifteen carrying at least one p.Ser1251Asn and nine carrying at least one (ultra)rare CFTR variant	LUMA IVA	Responses to CFTR modulators in organoids correlated with in vivo measurements (SCC and ppFEV_1_).	[50]
	Thirty-four children with CF carrying a wide range of CFTR variants	–	Organoid swelling significantly correlated with SCC and ICM.	[51]
	Thirty-four PwCF with p.Phe508del/p.Phe508del	–	Variability in CFTR residual function appeared to contribute to the clinical heterogeneity and organoid swelling values; responses in organoids correlated with ppFEV_1_ and BMI.	[52]
	Ninety-seven PwCF with well-characterized and rare CFTR variants	LUMA IVA	Measurements of residual CFTR function and rescue by CFTR modulators in organoids correlated with clinical data, namely changes in ppFEV_1_ and SCC.	[53]
	A 56-year-old female with CF carrying p.Phe508del/c.3717+5G>T	LUMA TEZA IVA	No baseline swelling was observed in organoids, suggesting minimal CFTR function; however, limited swelling was detected after LUMA/IVA or TEZA/IVA treatment.	[54]
	Twenty-one PwCF homozygous for p.Phe508del	LUMA IVA	No correlations were found between organoid swelling and changes in the in vivo biomarkers, namely SCC and NPD.	[55]
	A total of 173 PwCF carrying a wide range of CFTR variants	–	Organoid swelling values were associated with long-term ppFEV_1_ decline and the probability of developing different CF-related comorbidities, namely pancreatic insufficiency, CF-related liver disease, and CF-related diabetes.	[56]
	Fifteen PwCF homozygous for p.Phe508del, fifteen PwCF carrying p.Phe508del/class I variant, and twenty-two PwCF with rare variant non-eligible for CFTR modulator therapy	TEZA ELX IVA	Responses to modulators in organoids from p.Phe508del/p.Phe508del or p.Phe508del/class I variant correlated with changes in ppFEV_1_. In CF organoids with 11 rare genotypes, CFTR function restoration was reported upon ELX/TEZA/IVA treatment.	[57]
	A 19-year-old female with CF carrying p.Tyr515X/p.Arg334Trp	LUMA TEZA ELX IVA	Organoids treated with IVA alone or in combination with correctors demonstrated similar rescue of CFTR-dependent fluid secretion. In vivo measurements demonstrated significant clinical improvements in SCC, ppFEV_1,_ and respiratory symptoms after 7 days of IVA initiation that were sustained for the 9-month follow-up.	[58]
	A 6-year-old male with CF carrying p.Phe508del/p.Glu217Gly-Gly509Asp	LUMA TEZA ELX IVA	The p.Glu217Gly-Gly509Asp complex allele was characterized by a high residual function of the CFTR channel; organoid swelling values demonstrated rescue of CFTR function by all tested modulators.	[59]

Abbreviations: BMI—body mass index; CF—cystic fibrosis; CFTR—CF transmembrane conductance regulator; ELX—elexacaftor; HNE—human nasal epithelial; ICM—intestinal current measurement; IVA—ivacaftor; LUMA—lumacaftor; NPD—nasal potential differences; PwCF—people with CF; ppFEV_1_—percent predicted forced expiratory volume in 1 s; SCC—sweat chloride concentration; TEZA—tezacaftor.

**Table 2:** Studies assessing the effects of CFTR modulator therapies in CF inflammatory biomarkers.

CFTR Modulator(s)	Sample	CF Population/Genotypes	Key Findings	Ref.
**IVA**	Sputum	A total of 151 PwCF (≥6 years) carrying p.Gly551Asp	↓ bacterial load.	[94]
	Sputum	Three children with CF carrying p.Gly551Asp	No differences in airway microbiota density.	[95]
	Blood	Seven PwCF (≥6 years) carrying at least one residual function variant: p.Gln715X/p.Gly1349Asp; p.Glu1418ArgfsX14/p.Gly1349Asp; p.Gly1349Asp/p.Gly1349Asp; p.Ile1295PhefsX33/p.Gly1349Asp; p.Arg352AlafsX11/p.Gly1349Asp; p.Glu1418ArgfsX14/p.Ser549Arg; p.Phe508del/p.Gly1244Glu	↑ circulating mononuclear cell count after 2 months of IVA treatment.	[96]
	Sputum	Twelve PwCF (≥6 years) carrying p.Gly551Asp	↓ IL-8, IL-1β and NE levels; ↓ bacterial relative abundance; ↓ arginase-1, myeloperoxidase, calprotectin and S100A9.	[97]
	Blood/Sputum	Thirty-three PwCF (≥6 years) carrying p.Gly551Asp	Blood: ↓ CRP, IL-1β, IL-6, IL-8 and IL-10 levels. Sputum: ↓ bacterial relative abundance.	[98]
	Sputum	Thirty-one PwCF (≥6 years) carrying p.Gly551Asp	Despite improvements in SCC and ppFEV_1_, there were no significant changes in airway microbiota density, or in IL-6, IL-8, NE, IL-1β, SLPI, or A1AT levels.	[99]
	Epithelial lining fluid	Ten PwCF (≥6 years) carrying p.Gly551Asp	↓ IL-1β, IL-6, and IL-8 levels.	[100]
	Blood	Twenty PwCF carrying p.Gly551Asp	↓ white blood cell count and IL-6 and IL-8 levels.	[101]
	Blood	Ten PwCF (≥6 years) carrying p.Gly551Asp	↓ CXCL7, IL-8 and sTNFR1 levels.	[102]
	BALF	Thirty-nine children with CF carrying at least a gating variant	No differences in neutrophil count and free NE and IL-8 levels.	[103]
**LUMA/IVA**	Blood	Fourteen PwCF homozygous for p.Phe508del	No differences in white blood cell count or CRP levels.	[104]
	Blood/Sputum	Fourteen PwCF (≥16 years) homozygous for p.Phe508del	Blood: ↓ IL-8, IL-1β and TNF-α levels. Sputum: ↓ IL-6, IL-8, IL-1β and TNF-α levels.	[105]
	Sputum	Thirty PwCF (≥12 years) homozygous for p.Phe508del	↓ bacterial relative abundance and IL-1β levels.	[106]
	Sputum	Forty-one PwCF (≥12 years) homozygous for p.Phe508del	No differences in calprotectin levels or bacterial and fungal relative abundance.	[107]
**LUMA/IVA** **or** **TEZA/IVA**	Blood	LUMA/IVA: 13 PwCF homozygous for p.Phe508del TEZA/IVA: 8 PwCF homozygous for p.Phe508del	LUMA/IVA: ↓ IL-18, TNF-α, and caspase-1 levels. TEZA/IVA: ↓ IL-18, IL-1β, TNF-α, and caspase-1 levels.	[108]
**ELX/TEZA/IVA**	Blood	Forty-eight PwCF carrying at least one p.Phe508del	Of the cultures, 30% were negative to *P. aeruginosa*, and Meticilin-resistant *S. aureus*. ↓ IL-6, IL-8, IL-17A levels and neutrophil count.	[109]
	Sputum	Seventy-nine PwCF (≥12 years) carrying at least one p.Phe508del	↓ *P. aeruginosa* relative abundance, IL-1β, IL-8, NE, A1AT, and cathepsin-G levels. ↑ SLPI levels.	[110]
	BALF	Eight PwCF (≥12 years) carrying at least one p.Phe508del	↓ polymorphonuclear cell count. Bacterial cultures were negative for all subjects.	[111]
	Blood/Sputum	Thirty PwCF (≥12 years) carrying at least one p.Phe508del	Sputum: ↓ NE, PR3, cathepsin-G, IL-8, IL-1β levels and *P. aeruginosa* density. ↑ SLPI levels. Blood: ↓ IL-6, CRP, and A1AT levels.	[112]

Abbreviations: A1AT—alpha-1 antitrypsin; CF—cystic fibrosis; CRP—C-reactive protein; ELX—elexacaftor; IL—interleukin; IVA—ivacaftor; LUMA—lumacaftor; NE—neutrophil elastase; ppFEV1—percent-predicted forced expiratory volume in 1 s; SCC—sweat chloride concentration; SLPI—secretory leukocyte protease inhibitor; sTNFR—soluble tumor necrosis factor receptor; TEZA—tezacaftor.

**Table 3:** Studies reporting microRNAs involved in CFTR regulation and CF inflammatory process.

MicroRNA	Target	Key Mechanism(s)	Ref.
miR-17	IL-8	*↓* IL-8 levels *P. aeruginosa* infection reduced miR-17 levels	[143]
miR-31	IRF-1	*↓* cathepsin-S production	[144]
miR-93	IL-8	*P. aeruginosa* infection reduced miR-93 levels and increased IL-8 production	[145]
miR-126	TOM1	↑ IL-8 levels and NF-κB signaling	[146]
miR-145	CFTR SMAD3	Repression of CFTR 3′-UTR reporter *↓* TGF-β signaling	[147,148,149] [147,150]
miR-155	SHIP1	*↓* SHIP1 levels ↑ IL-8 levels and PI3K/Akt signaling	[151]
miR-199a-5p	CAV1	*↓* CAV1 levels ↑ TLR4 signaling	[152]
miR-223	CFTR	Repression of CFTR 3′-UTR reporter	[148]
miR-384	CFTR and SLC12A2	*↓* CFTR and SLC12A2 expression levels	[149]
miR-494	CFTR and SLC12A2	*↓* CFTR and SLC12A2 expression levels Repression of CFTR 3′-UTR reporter ↑ NF-κB signaling, TNF-α, and IL-1β levels increased miR-494 activity	[149] [147,148,149] [153]
miR-509-3p	CFTR	↑ NF-κB signaling, TNF-α, and IL-1β levels increased miR-494 activity	[153]
miR-1246	CFTR and SLC12A2	*↓* CFTR and SLC12A2 expression levels	[149]

Abbreviations: CFTR—cystic fibrosis transmembrane conductance regulator; IL—interleukin; IRF—interferon regulatory factor; NF-κB—nuclear factor κB; SLC—solute carrier; TLR—Toll-like receptor; TGF—transforming growth factor; TNF—tumor necrosis factor.

## Abbreviations

ALI—air–liquid interface; BALF—bronchoalveolar lavage fluid; CF—cystic fibrosis; CFTR—CF transmembrane conductance regulator; CRP—C-reactive protein; ELX—elexacaftor; EMA—European Medicines Agency; EV—extracellular vesicle; FDA—Food and Drug Administration; FIS—forskolin-induced swelling; HBE—human bronchial epithelial; HMGB—high-mobility group box; HNE—human nasal epithelial; ICM—intestinal current measurement; IL—interleukin; IVA—ivacaftor; LUMA—lumacaftor; miR—microRNA; NE—neutrophil elastase; NPD—nasal potential difference; PM—plasma membrane; ppFEV_1_—percent predicted forced expiratory volume in one second; PwCF—people with CF; ROMA—rectal organoid morphology analysis; SCC—sweat chloride concentration; SLA—steady-state lumen area; TEZA—tezacaftor; TNF—tumor necrosis factor.
